# Patient Perception of the Use of the Apple Vision Pro Virtual Reality Headset in an Anesthesiology Informed Consent Process: Prospective Randomized Controlled Crossover Trial

**DOI:** 10.2196/95806

**Published:** 2026-07-23

**Authors:** Sabrina Chriqui, John Francis Ryan, Bao Kien Luu, Minh Tran, Byron Fergerson, Alan You, Christian Dameff, Preetham John Suresh, Jeffrey Logan Tully

**Affiliations:** 1 School of Medicine, University of California San Diego, 9500 Gilman Dr, La Jolla, CA, 92093, United States, 1 (858) 534-0830; 2 Department of Anesthesiology and Pain Medicine, University of California Davis Health, Sacramento, CA, United States; 3 Department of Surgery, University of California San Diego Health, La Jolla, CA, United States; 4 Department of Anesthesiology, University of California San Diego Health, La Jolla, CA, United States; 5 Division of Clinical Informatics and Telehealth, Department of Emergency Medicine, University of California San Diego Health, La Jolla, CA, United States; 6 Department of Medicine, University of California San Diego Health, La Jolla, CA, United States; 7 Department of Biomedical Informatics, Department of Medicine, University of California San Diego Health, La Jolla, CA, United States; 8 Department of Computer Science and Engineering, Jacobs School of Engineering, University of California San Diego, La Jolla, CA, United States; 9 Division of Perioperative Informatics, Department of Anesthesiology, University of California San Diego Medical Center, La Jolla, CA, United States

**Keywords:** Apple Vision Pro, augmented reality, virtual reality, compassion, headset, Sinclair Compassion Questionnaire

## Abstract

**Background:**

Augmented reality (AR) and virtual reality (VR) have become mainstream commercial technologies, with AR integrating virtual elements into displays of reality and VR offering fully immersive computer-generated visual experiences. The Apple Vision Pro is a VR headset with an outward-facing display that depicts the user’s eyes to the outside world. This headset is actively incorporated into patient care, but questions remain about the impact of such a device on the patient-physician relationship.

**Objective:**

This study surveyed patients’ responses to the use of an Apple Vision Pro VR headset by anesthesiologists during an informed consent discussion for sedation for endoscopic gastrointestinal procedures. We sought to qualify the impact, if any, of the Apple Vision Pro on patients’ perceptions of connectedness with their anesthesiologist, including compassion and empathy felt from their physician.

**Methods:**

This prospective randomized controlled crossover study involved 60 patients undergoing sedation for gastrointestinal procedures at the University of California, San Diego Health’s Hillcrest Medical Center and Jacobs Medical Center. An anesthesiologist had 2 encounters with each patient prior to their procedure, 1 while the anesthesiologist wore an Apple Vision Pro and 1 without wearing any headset. After both encounters, patients completed a standardized, validated survey regarding the compassion and empathy they felt from the anesthesiologist during each encounter. They also completed a custom survey with additional questions related to their perception of technology in health care and their own personal prior experience with AR and VR.

**Results:**

While 16.7% (10/60) of the participants owned an AR or VR headset of their own, 73% (44/60) had no prior experience with AR or VR. Participants reported consistently high ratings across all empathy and compassion measures for both the with-headset and without-headset conditions; however, mean Sinclair Compassion Questionnaire scores were statistically significantly higher for the no-headset condition (4.69, SD 0.48) when compared to the with-headset condition (4.42, SD 0.87; *P*=.003). Participants reported significantly reduced ability to focus on their conversation with the anesthesiologist when they were wearing the Apple Vision Pro (*P*<.001), with a large effect size (*r*=0.614). The most pronounced difference was observed with regard to patients’ satisfaction with eye contact made with their physician, which decreased significantly when they wore the Apple Vision Pro (*P*<.001; large effect size: *r*=0.736).

**Conclusions:**

This study examined the impact of physicians wearing the Apple Vision Pro during patient interactions, revealing a statistically significant reduction in patients’ perceived compassion from and connectedness with their anesthesiologist. This highlights the need for caution when integrating AR or VR technology into clinical practice as it may interfere with the patient-physician relationship. Careful evaluation of the potential effects on patients’ trust in their physician is essential before adopting AR or VR in health care settings.

## Introduction

The integration of augmented reality (AR) and virtual reality (VR) headsets into clinical practice is a rapidly developing objective across research and industry [1-3]. AR refers to the use of devices that display views of the real world that are modified or enhanced with superimposed computer-generated graphics [4]. VR refers to the use of devices in which the visual field is composed solely of computer-generated graphics [5]. In the last decade, technology companies have made relatively affordable head-mounted displays available to the public, thus making AR and VR increasingly accessible to the general consumer. As such, these technologies are being rapidly integrated into medical training and clinical practice. For example, AR has been used to improve surgical education and intraoperative guidance for surgeons, with multiple studies using the technology to improve operative efficiency and outcome measures in neurosurgery, orthopedics, oncology, ophthalmology, and otolaryngology [6-11]. Additionally, AR and VR have been studied in the context of patients wearing a head-mounted device for the purposes of rehabilitation, patient education, and even distraction [12]. However, the use of AR and VR in patient care by clinicians outside of the operative or procedural setting remains largely unexplored.

In June 2023, the release of the Apple Vision Pro by Apple Inc appeared to represent a paradigm shift in the intersection of AR and VR and medicine. Clinicians and researchers alike rushed to take advantage of the device’s increased processing power, futuristic hand-eye tracking, and realistic “pass-through” vision [13-19]. Additionally, major health care players began exploring the use of the Apple Vision Pro in more everyday clinical settings, such as clinicians using the headset to augment documentation in the electronic health record during face-to-face patient interactions. Epic Systems Corporation introduced its Epic Spatial Computing Concept application for the Apple Vision Pro and announced a partnership with Sharp HealthCare to create the Spatial Computing Center of Excellence [20-22]. Ultimately, the Apple Vision Pro has inspired a departure from the more well-studied use of AR and VR during non–patient-facing encounters. Now, the prospect of clinicians entering face-to-face patient interactions wearing an eye-occluding head-mounted display is becoming increasingly realistic, raising an important question: How would patients perceive their physician wearing an AR or VR headset during an encounter?

A strong physician-patient relationship is the cornerstone of effective health care as this impacts patient satisfaction, adherence to treatment plans, and overall health outcomes [23]. At the core of this relationship is the physician’s ability to express empathy, establish trust, and communicate effectively. Eye contact is one of the most important nonverbal factors affecting perceived physician empathy, even more so than social touch or encounter length [24,25]. As such, it is reasonable to question whether a physician wearing an AR or VR headset would hinder patients’ perception of empathy and/or compassion from that physician.

Previous studies have demonstrated that certain AR headsets with clear lenses, which still allow for natural eye contact to be made between the physician and patient, do not necessarily negatively impact the patient-physician relationship. For example, one study taking place in an outpatient dermatology clinic found that patients gave physicians wearing the Google Glass smart glasses high scores on questions evaluating their relationship with their physician (9.4/10, SD 0.93) and their physician’s communication with them (9.5/10, SD 1.10). A total of 87.1% of these patients were somewhat or extremely comfortable with their physician wearing the smart glasses, and 94.7% said that they were equally or more likely to trust their physician while they were wearing the Google Glass [26]. However, the impact of completely occlusive VR headsets on eye contact and patients’ perception of compassion and empathy has not been studied. Although the Apple Vision Pro is technically a completely occlusive VR headset, it does feature an external display that projects a virtual representation of the wearer’s eyes, which Apple calls EyeSight. This feature aims to keep users connected with those around them by simulating real-world eye contact [27].

In this prospective crossover trial, we investigated how a physician wearing the Apple Vision Pro headset with EyeSight impacted the patient-physician relationship by assessing patients’ perceptions of empathy, compassion, and communication when speaking to a physician wearing the Apple Vision Pro. Understanding these perceptions is essential for ensuring that emerging technologies enhance rather than hinder patient-centered care.

## Methods

### Study Design and Setting

This prospective randomized controlled crossover study took place in the gastrointestinal (GI) endoscopy unit’s preprocedural areas at the University of California, San Diego Health’s Jacobs Medical Center and Hillcrest Medical Center between September 2024 and December 2024. Three attending anesthesiologists (authors BF, MT, and JLT) approached patients scheduled for endoscopic GI procedures and obtained informed consent for monitored anesthesia care 2 times, once without wearing a headset and once while wearing the Apple Vision Pro. A survey was administered to the patient after each encounter to evaluate their perception of their anesthesiologist’s compassion and empathy, connectedness, communication, and eye contact.

### Participants

This study used a convenience sample of patients scheduled for nonemergent endoscopic GI procedures at the University of California, San Diego Health. Inclusion criteria required that patients be at least 18 years of age and able to converse, read, and write in English. Exclusion criteria were patients belonging to the following vulnerable populations: children under 18 years of age, pregnant individuals, incarcerated individuals, and those with an unhoused status. Also excluded were patients undergoing an emergent procedure or who, based on the judgment of the anesthesiologist, lacked capacity to provide informed consent to participate in the study. Participants who completed the study protocol were compensated for their time and effort with a US $5 gift card.

### Randomization and Study Protocol

Each participant completed 2 consecutive informed consent encounters: 1 in which the physician did not wear the Apple Vision Pro headset and 1 in which the physician wore the headset. Participants were randomly assigned to 1 of 2 encounter sequences (with the headset first or without the headset first). In both sequences, an anesthesiologist conducted a standard preprocedure evaluation including medical history, physical examination, and informed consent discussion for monitored anesthesia care. Every participant then underwent a repeat informed consent discussion under the alternate condition. Both encounters were conducted sequentially, with only a 30-second interval between them; the physician briefly exited and then re-entered the patient area to either put on the headset or remove it. When the Apple Vision Pro headset was used first, it was removed during the physical examination section to allow for an adequate clinical assessment. Although sequence order was randomized to minimize order effects, sequence assignment was not recorded for individual participants. Participants were informed before the encounters that the study aimed to assess patient perceptions of physician communication with and without the use of an AR or VR headset. They were not told that the headset was expected to improve clinical care but were made aware that it is an established technology currently used in health care settings and that the study sought to understand their perspectives on its use.

### Technology Implementation

**Figure 1.**
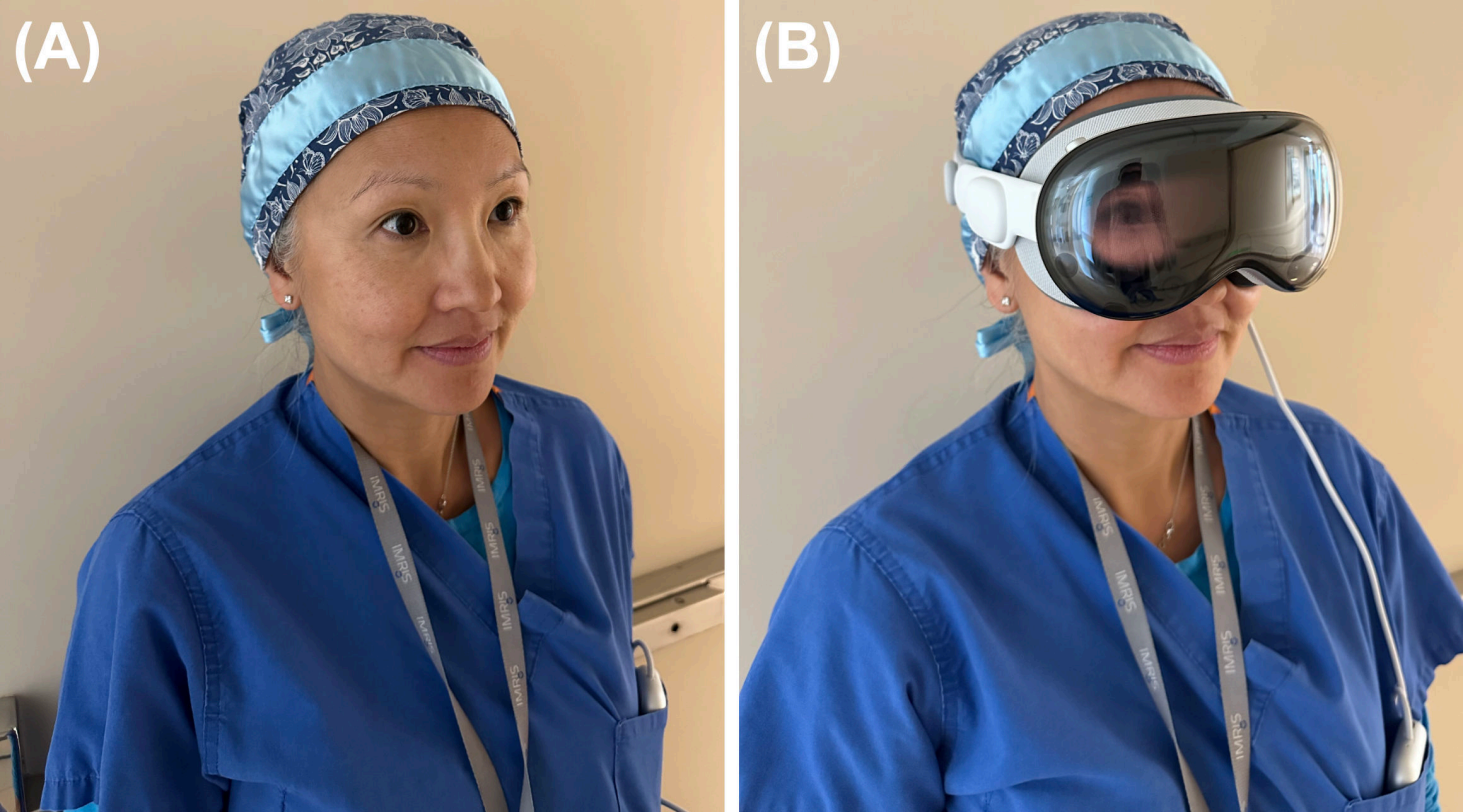
EyeSight displays a representation of the wearer’s eyes on the Apple Vision Pro’s outward-facing display. This figure depicts an anesthesiologist (author MT) before (A) and after (B) donning the Apple Vision Pro headset. The wearer must undergo a brief facial scan to activate EyeSight. The representation of the wearer’s eyes may vary depending on the location of the viewer and the angle at which they are viewing the outward-facing display.

Prior to patient encounters, the Apple Vision Pro headset was configured to display a realistic representation of the physician’s eyes using the device’s built-in face scanning technology (Figure 1). This feature was implemented to maintain a simulacrum of eye contact during patient interactions.

### Data Collection

Nonidentifying demographic data were obtained from each participant, including their age, gender, and race and ethnicity, as well as basic questions regarding their prior experience with AR and VR, if any. Immediately following both encounters, patients completed the Sinclair Compassion Questionnaire (SCQ), a validated 15-item instrument created by Sinclair et al [28,29] that measures patient-perceived compassion in health care encounters and includes assessments of clinician attentiveness, empathy, and caring behaviors. Each item was rated on a 5-point Likert scale ranging from “strongly disagree” (1) to “strongly agree” (5). The SCQ is scored by calculating the mean score of all items, with higher scores indicating greater reported compassion. Patients completed the SCQ once for each encounter (headset and no headset). In addition, the patients completed a separate questionnaire (Multimedia Appendix 1) more specific to this study that assessed, from the patient perspective, patients’ and physicians’ ability to focus during each conversation, their satisfaction with eye contact made, how realistic EyeSight felt, and how concerned they were about their privacy during each encounter. They also responded to 5 questions assessing their general beliefs about technology in health care, whether AR or VR can improve health care delivery, and whether informed consent should be required from patients prior to using AR or VR during clinical encounters.

### Statistical Analysis

Survey responses were analyzed to compare patient perceptions between the with-headset and without-headset conditions. Due to the paired nature of the crossover design and the nonnormal distribution of the Likert-scale responses, the Wilcoxon signed-rank test was used to compare SCQ scores and individual survey items between conditions [30]. Effect sizes were calculated as *Z*/√*N*, where *Z* is the standardized test statistic and *N* is the number of paired observations, and interpreted according to the criteria by Cohen [31] (small: *r*=0.1; medium: *r*=0.3; large *r*=0.5). All statistical analyses were conducted using R (version 4.3.1; R Foundation for Statistical Computing). All *P* values were 2 tailed, and a *P* value of less than .05 was considered significant.

### Ethical Considerations

The study protocol was reviewed by the director of the Office of Institutional Review Board (IRB) Administration, IRB chair, or IRB chair’s designee at the University of California, San Diego, and was determined to be exempt from institutional review (810467) under Title 45 of the Code of Federal Regulations, part 46.104(d) [32].

## Results

### Demographic Characteristics

This study included 60 participants with a mean age of 59 (SD 15) years, ranging from 18 to 91 years (Table 1). A total of 53.3% (n=32) of the participants were female. The racial and ethnic breakdown was 80% (n=48) White, 8.3% (n=5) Asian, 6.7% (n=4) Hispanic or Latino, and 5% (n=3) Black. Approximately 73% (n=44) of the participants had no prior experience with AR or VR, and only 16.7% (n=10) owned an AR or VR headset.

**Table 1.** Overview of participant demographics. As part of the initial survey, patients voluntarily provided their self-identified gender, age, and race and ethnicity (N=60).

Demographic	Participants, n (%)
Gender	
Women	32 (53.3)
Men	28 (46.7)
Age (y)	
29	1 (1.7)
30-39	6 (10)
40-49	12 (20)
50-59	11 (18.3)
60-69	15 (25)
70-79	13 (21.7)
80-89	1 (1.7)
≥90	1 (1.7)
Race and ethnicity	
Asian	5 (8.3)
Black or African American	3 (5)
Hispanic or Latino	4 (6.7)
Native Hawaiian or other Pacific Islander	0 (0)
White	48 (80)
Other	0 (0)

### Physician Empathy and Compassion Satisfaction Scores

Participants reported consistently high ratings across all empathy and compassion measures for both with-headset and without-headset conditions; however, mean SCQ scores were statistically significantly higher for the without-headset condition (4.69, SD 0.48) when compared to the with-headset condition (4.42, SD 0.87) using the Wilcoxon signed-rank test (*P*=.003 [28,29]).

### Paired Comparison of Custom Questions

The difference between responses to custom survey questions in the headset and no-headset conditions are shown in Table 2. Participants reported significantly reduced ability to focus on their conversation with the anesthesiologist when they were wearing the Apple Vision Pro (*P*<.001), with a large effect size (*r*=0.614). Patients also perceived that their anesthesiologist was less focused while wearing the Apple Vision Pro (*P*<.001), with a medium effect size (*r*=0.454). The most pronounced difference was observed with regard to patients’ satisfaction with eye contact made with their physician, which decreased significantly when they wore the Apple Vision Pro (*P*<.001; large effect size: *r*=0.74). While statistically significant differences were observed between conditions, overall ratings across both conditions remained generally high.

**Table 2.** Summary and statistical analysis of participants’ responses to survey statements comparing their interactions with the physician with and without the Apple Vision Pro headset^a^.

Survey statement (abbreviated)	No headset, median rating	With headset, median rating	*P* value	Effect size	Effect classification
I was able to focus on the conversation.	Strongly agree	Agree	<.001	0.614	Large
My physician was able to focus on the conversation.	Strongly agree	Strongly agree	<.001	0.454	Medium
I was satisfied with my ability to make eye contact.	Strongly agree	Agree	<.001	0.736	Large
I was concerned about my privacy.	Strongly disagree	Strongly disagree	.002	0.416	Medium
The portrayal of the physician’s eyes was realistic.	—^b^	Agree	—	—	—
If the headset was completely occlusive, I would be opposed to my physician wearing it.	—	Neutral	—	—	—

a Responses were recorded on a 5-point Likert scale (“strongly disagree” to “strongly agree”). Paired patient responses were evaluated for statistical significance using the Wilcoxon signed-rank test. Effect sizes were interpreted according to the criteria by Cohen [31].

b Asked only in the headset condition; no without-headset comparison was available, so these items are reported descriptively.

**Figure 2.**
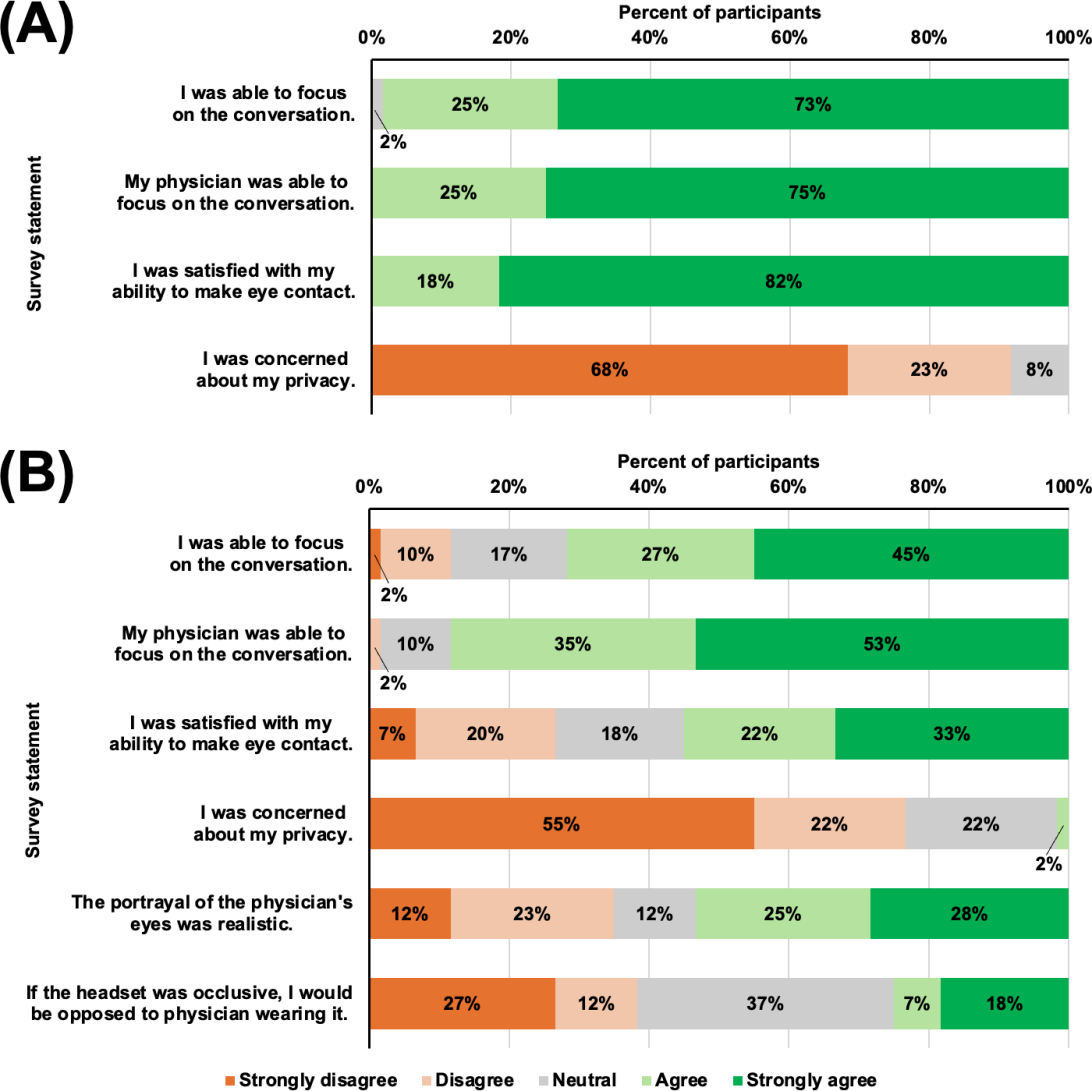
Patient responses to custom survey questions regarding their experience with an anesthesiologist under traditional circumstances (A) vs while wearing an Apple Vision Pro headset (B). The figure depicts Likert-scale data, with participants rating each statement from “strongly disagree” to “strongly agree.” These responses were statistically analyzed, the results of which are presented in Table 2.

Regarding the Apple Vision Pro’s EyeSight feature, 53.3% (32/60) of the patients agreed or strongly agreed that the representation of the physician’s eyes on the outward-facing display was realistic, whereas 35% (21/60) disagreed or strongly disagreed. One-quarter (15/60, 25%) of patients reported that they would be opposed to their physician wearing the headset if it were completely occlusive and did not allow for simulated eye contact (Figure 2).

### Technology Acceptance and Privacy

**Figure 3.**
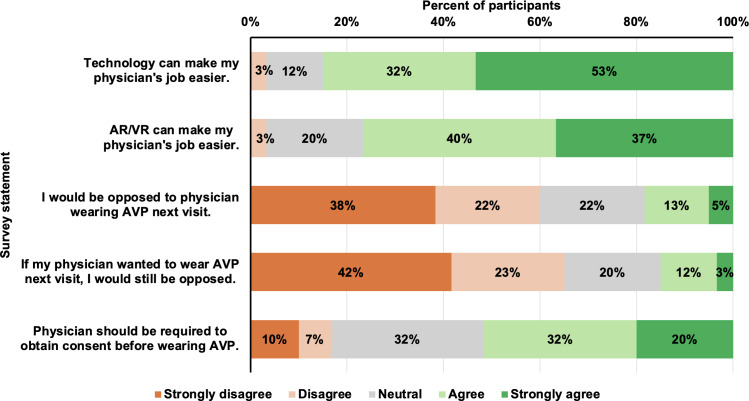
Patient perspectives on the role of augmented reality (AR) and virtual reality (VR) in health care and the need for informed consent from patients in these situations. Participants responded to each statement on a Likert scale ranging from “strongly disagree” to “strongly agree.” AVP: Apple Vision Pro.

Custom questions regarding the patients’ overall opinions on the role of technology in health care revealed high levels of technology acceptance (Figure 3). In total, 85% (51/60) of the patients agreed or strongly agreed that technology can make a physician’s job easier, with 76.7% (46/60) agreeing that AR or VR can make a physician’s job easier during patient encounters. A total of 18.3% (11/60) of the patients were opposed or strongly opposed to their physician wearing an AR or VR headset at their next encounter. When the question was slightly changed to ask whether they would still be opposed if their physician told them they preferred to use it, 13.3% (8/60) of the patients were opposed.

Just over half (31/60, 51.7%) of participants believed that physicians should be required to obtain consent from patients before wearing an AR or VR headset during an encounter. Patients reported slightly increased privacy concerns during the headset encounter compared to no headset (*P*=.002; *r*=0.42); however, this was primarily caused by an increased number of patients responding neutrally (3 out of 5) to the following statement: “I was concerned about my privacy.” One patient in the headset condition agreed with the statement, and no patients strongly agreed.

## Discussion

### Principal Findings

We found that the use of an AR or VR headset negatively impacted perceptions of provider empathy and communication. While participants in both conditions reported relatively high SCQ ratings, the with-headset encounters received statistically significantly lower SCQ scores than the without-headset encounters, suggesting that the technology may create subtle barriers to physician-patient connection. We hypothesize that this decline in perceived compassion could stem from reduced eye contact, altered nonverbal communication, or a sense of detachment caused by the headset’s presence.

EyeSight received mixed reviews from participants in this study as just over half of patients found EyeSight to be realistic (32/60, 53%) and felt satisfied with their ability to maintain eye contact with their physician (33/60, 53%). Although we did not evaluate a fully occlusive headset in our study, 25% (15/60) of the participants reported that they would be opposed to their physician wearing one. Our findings are relatively consistent with those of Vergari et al [33], who conducted a pilot study showing that interactions with the Apple Vision Pro were perceived as more engaging and comprehensible compared to those using the Meta Quest 3, a fully occlusive headset, but less than face-to-face interactions.

The findings of the technology acceptance questionnaire suggest that patients remain open to the use of technology in health care workflows, with most patients recognizing its potential to enhance physician efficiency and health care delivery. Specifically, over three-quarters of the participants agreed that technology in general can improve health care delivery (51/60, 85%) and that AR and VR specifically could enhance physician capabilities (46/60, 77%).

Despite this overall acceptance, some reservations remain regarding the direct implementation of AR and VR headsets in patient encounters. A subset of patients opposed physician use of the headset, and over half (31/60, 51.7%) of patients believed that physicians should obtain consent before wearing the headset during an appointment, highlighting the importance of maintaining patient autonomy in technology integration. Notably, privacy concerns were minimal, with only 1.7% (1/60) of the patients expressing concern while the physician used the Apple Vision Pro. These findings underscore the need for clear communication between physicians and patients regarding the purpose and benefits of AR and VR technology while also ensuring that workflows integrating this technology incorporate patient preferences and privacy-preserving frameworks.

This study offers several insights into how AR technology may be more effectively integrated into clinical encounters. As 16.7% (10/60) of the patients would be opposed to their physician wearing the Apple Vision Pro at their next visit and 25% (15/60) would be opposed to their physician wearing a fully occlusive headset, the simulated eye contact provided by the EyeSight feature may be slightly preferable to a fully occlusive headset; however, it likely does not fully preserve the benefits of natural eye contact [33]. Finally, the justification for physician use of these technologies, including measured or perceived benefits, and an acknowledgment of the potential for friction in the patient-physician relationship should be integrated into larger discussions of informed consent. Such transparency may reduce patient confusion or discomfort, allowing the technology to be a supportive element of care rather than a distraction.

While the Apple Vision Pro certainly holds potential to enhance physician workflows and patient care, we believe that its success may depend on thoughtful design, clinician preparation, and open communication with patients.

### Limitations

This was a single-center study at an academic institution, which may limit the generalizability of these results. Participant demographics were skewed relative to the local patient population (eg, 48/60, 80% of the participants identified as White). We studied a convenience sample of patients undergoing endoscopic GI procedures due to the high turnover of patients in one day and the relatively lower acuity of cases; however, this likely skewed the age range of our participants (53/60, 88.3% were over 40 years of age, and 30/60, 50% were over 60 years of age). Generalizability is also limited by our study evaluating brief preprocedural encounters; these results may not be generalizable to longer patient encounters, such as in a primary care clinic. The short interval between the headset and no-headset interactions may have introduced a carryover effect into our data as there was no washout period. Finally, the novelty effect associated with new technologies warrants further longitudinal studies to assess whether reactions may normalize over time as adoption increases.

### Future Directions

Future research should build on this study by incorporating a larger and more diverse patient population to better understand how patients of different ages and from different backgrounds perceive the use of AR and VR in health care. Comparing additional headset designs—including AR glasses (eg, Meta Orion), an AR headset with transparent lenses (eg, Microsoft HoloLens), or a fully occlusive VR headset (eg, Meta Quest)—could help determine which type of design best balances physician needs and patient comfort with or trust in their physician. Additionally, further investigation is needed to develop strategies that minimize perceived reductions in physician attention and empathy. Future studies should also more deeply assess AR and VR’s influence on the physician-patient relationship over time by including validated scales of therapeutic alliance and communication. Finally, long-term studies are also needed to follow how patient opinions change over time as availability, affordability, and adoption of this technology increases.

### Conclusions

As AR and VR technologies are adopted into clinical settings, their impact on the empathy and communication from physicians must be carefully considered. Statistically significantly lower SCQ scores in the headset condition suggest that, without thoughtful design and implementation, AR and VR headsets may introduce unintended barriers to patient focus and engagement. However, patients remain open-minded with regard to the implementation of technology, including AR and VR, into clinical practice, and most would not oppose its use if it helps their physician perform their job better or more easily. Careful implementation and ongoing research will be essential to fully realize the potential of AR and VR in clinical settings while ensuring that patient-centered care and high-quality communication remain a top priority.

## Supplementary material

10.2196/95806 Multimedia Appendix 1 Custom survey questions answered by participants.

## Abbreviations

AR: augmented reality
GI: gastrointestinal
IRB: Institutional Review Board
SCQ: Sinclair Compassion Questionnaire
VR: virtual reality
