# Supplementary material for: Patient Perception of the Use of the Apple Vision Pro Virtual Reality Headset in an Anesthesiology Informed Consent Process: Prospective Randomized Controlled Crossover Trial
Source: JMIR XR Spat Comput. 2026 Jul 23;3:e95806. doi: 10.2196/95806 (PMC13397004; doi:10.2196/95806)
Supplement: Multimedia Appendix 1 [file xr-v3-e95806-s001.docx]

**Appendix**

**Appendix 1. Custom survey questions answered by participants.**

The following questions were asked twice, once while the participant considered their experience with the physician wearing the AVP, and once while they considered their experience with the physician not wearing a headset. Participants responded to each statement on a Likert scale ranging from “Strongly Disagree” to “Strongly Agree.”

I was able to focus on my conversation with my Healthcare Provider.

My Healthcare Provider appeared to be focused on their conversation with me.

I was satisfied with my ability to make eye contact with my Healthcare Provider.

I was concerned about my privacy during the encounter.

The following questions were asked once while the participant considered their experience with the physician wearing the AVP. Participants responded to each statement on a Likert scale ranging from “Strongly Disagree” to “Strongly Agree.”

The portrayal of my Healthcare Provider’s eyes was realistic.

If the headset was completely occlusive (i.e., I could not see the Healthcare Provider’s eyes at all), I would be opposed to my Healthcare Provider wearing it during the encounter.

The following questions were answered by all participants one time. Participants responded to each statement on a Likert scale ranging from “Strongly Disagree” to “Strongly Agree” unless otherwise stated.

I believe that, in general, technology can make my Healthcare Provider’s job easier during patient encounters.

I believe that, in general, augmented reality (AR) or virtual reality (VR) can make my Healthcare Provider’s job easier during patient encounters.

If it were up to me, I would be opposed to my Healthcare Provider wearing an AR/VR headset at my next visit.

If my Healthcare Provider preferred to wear an AR/VR headset during my next visit, I would still be opposed to them wearing it.

Healthcare Providers should be required to obtain consent from patients prior to wearing an AR/VR headset during an encounter.

What is your age? (Options: Numbers 18-89, 90+)

What is your gender? (Options: Male, Female, Non-binary, Other)

What is your race and/or ethnicity? (Options: American Indian or Alaska Native, Asian, Black or African American, Hispanic or Latino, Native Hawaiian or Other Pacific Islander, White, Other)

Have you ever used an augmented reality (AR) or virtual reality (VR) headset? If so, which one(s)? (Options: No, I have never used an AR/VR headset; Yes, but I am unsure of which one(s); Apple Vision Pro; Meta Quest; Microsoft HoloLens; PlayStation VR; Valve Index; Other)

Do you own, or have you ever owned, an augmented reality (AR) or virtual reality (VR) headset? If so, which one(s)? (Options: No, I have never owned an AR/VR headset; Yes, but I am unsure of which one(s); Apple Vision Pro, Meta Quest, Microsoft HoloLens, PlayStation VR; Valve Index; Other)

On average, how many days per month do you use an AR/VR headset? (Regardless of whether or not you own the headset) (Options: Fill-in-the-blank)
